# Brain serotonin and serotonin transporter expression in male and female postnatal rat offspring in response to perturbed early life dietary exposures

**DOI:** 10.3389/fnins.2024.1363094

**Published:** 2024-03-21

**Authors:** Xin Ye, Shubhamoy Ghosh, Bo-Chul Shin, Amit Ganguly, Liesbeth Maggiotto, Jonathan P. Jacobs, Sherin U. Devaskar

**Affiliations:** ^1^Department of Pediatrics, Division of Neonatology & Developmental Biology and The Neonatal Research Center of the Children's Discovery & Innovation Institute, David Geffen School of Medicine at UCLA, Los Angeles, CA, United States; ^2^The Vatche and Tamar Manoukian Division of Digestive Diseases, David Geffen School of Medicine at UCLA, Los Angeles, CA, United States; ^3^Division of Gastroenterology, Hepatology and Parenteral Nutrition, Veterans Affairs Greater Los Angeles Healthcare System, Los Angeles, CA, United States

**Keywords:** serotonin, serotonin transporter, caloric restriction, intrauterine growth restriction, high fat diet, microbiome

## Abstract

**Introduction:**

Serotonin (5-HT) is critical for neurodevelopment and the serotonin transporter (SERT) modulates serotonin levels. Perturbed prenatal and postnatal dietary exposures affect the developing offspring predisposing to neurobehavioral disorders in the adult. We hypothesized that the postnatal brain 5-HT-SERT imbalance associated with gut dysbiosis forms the contributing gut-brain axis dependent mechanism responsible for such ultimate phenotypes.

**Methods:**

Employing maternal diet restricted (IUGR, n=8) and high fat+high fructose (HFhf, n=6) dietary modifications, rodent brain serotonin was assessed temporally by ELISA and SERT by quantitative Western blot analysis. Simultaneously, colonic microbiome studies were performed.

**Results:**

At early postnatal (P) day 2 no changes in the IUGR, but a ~24% reduction in serotonin (*p* = 0.00005) in the HFhf group occurred, particularly in the males (*p* = 0.000007) revealing a male versus female difference (*p* = 0.006). No such changes in SERT concentrations emerged. At late P21 the IUGR group reared on HFhf (IUGR/HFhf, (*n* = 4) diet revealed increased serotonin by ~53% in males (*p* = 0.0001) and 36% in females (*p* = 0.023). While only females demonstrated a ~40% decrease in serotonin (*p* = 0.010), the males only trended lower without a significant change within the HFhf group (*p* = 0.146). SERT on the other hand was no different in HFhf or IUGR/RC, with only the female IUGR/HFhf revealing a 28% decrease (*p* = 0.036). In colonic microbiome studies, serotonin-producing Bacteriodes increased with decreased Lactobacillus at P2, while the serotonin-producing Streptococcus species increased in IUGR/HFhf at P21. Sex-specific changes emerged in association with brain serotonin or SERT in the case of *Alistipase, Anaeroplasma, Blautia, Doria, Lactococcus*, Proteus, and *Roseburia genera*.

**Discussion:**

We conclude that an imbalanced 5-HT-SERT axis during postnatal brain development is sex-specific and induced by maternal dietary modifications related to postnatal gut dysbiosis. We speculate that these early changes albeit transient may permanently alter critical neural maturational processes affecting circuitry formation, thereby perturbing the neuropsychiatric equipoise.

## 1 Introduction

Serotonin (5-HT) is a neurotransmitter in the brain. Among the multiple roles of serotonin in the brain, the most intriguing is its developmental role that has been recognized in recent years (Gaspar et al., 2003; Sodhi and Sanders-Bush, 2004; Daws and Gould, 2011). Serotonin modulates multiple physiological functions such as gastrointestinal motility, cardiovascular function, memory and mood in adult humans. The role of serotonin in neuropsychiatric conditions, such as autism spectrum or anxiety–related disorders, led to the recognition of a role during development (Whitaker-Azmitia et al., 1996; Whitaker-Azmitia, 2001, 2005; Gaspar et al., 2003; Daws and Gould, 2011).

Embryonic and postnatal mammalian brain development is highly reliant on multiple factors, one of which is serotonin. During embryonic and postnatal development, serotonin modulates neurodevelopmental processes, such as neuron cell division, migration and differentiation, axonal and dendritic elaboration, connectivity and myelination (Gaspar et al., 2003). Serotonergic neurons are first detected in the 5-week-old human embryos exhibiting rapid growth and proliferation (Sundstrom et al., 1993). After 15 weeks, serotonin neuron somata clusters are seen in raphe nuclei. In rodents, serotonin neurons appear at ~10.5 days gestation and begin releasing serotonin at ~13 days (E13) (Takahashi et al., 1986). Postnatally, brain serotonin concentrations peak in the first week of life, only to decline thereafter reaching adult concentrations at ~postnatal day 15 (P15) (Daws and Gould, 2011). Serotonin neurons continue their axonal growth through postnatal life until P21 (Daws and Gould, 2011). Perturbed brain serotonin concentrations during critical windows of development have long-lasting persistent effects on brain function, contributing toward the subsequent development of altered behaviors resulting in anxiety/depression related conditions during adult life (Whitaker-Azmitia, 2001; Sodhi and Sanders-Bush, 2004).

Serotonin transporter (SERT or 5-HTT) is a major regulator of serotonin neurotransmission and it has been implicated in the etiology of autism spectrum disorders (ASDs) (Muller et al., 2016). In the mature animal, SERT is located on presynaptic serotonin nerve terminals and serotonin neuronal cell bodies (Daws and Gould, 2011). SERT is first expressed during prenatal rodent brain development at E12, and by E18 it is found in all subcellular neuronal compartments (Bruning et al., 1997; Daws and Gould, 2011). During the course of embryonic development, SERT density peaks with advancing gestation (Moll et al., 2000; Daws and Gould, 2011) and is reported to be highest at P14, declining to relatively stable concentrations in adolescent rodents. This temporal pattern of SERT follows that seen with serotonin by one postnatal week of age. During critical stages of brain development, serotonin concentrations can influence the expression or function of key regulators of serotonin neurotransmission, including SERT (Brummelte et al., 2017). SERT expression and function are influenced by a large and diverse array of intrinsic and extrinsic factors. Intrinsic factors include genetic variants, auto- and hetero-dimerization, cytokines and hormones. Extrinsic factors such as diet, environmental stressors and drugs can also have prominent effects on SERT expression and function. Less is known about how these factors might come into play during prenatal and postnatal critical phases of development (Daws and Gould, 2011; Brummelte et al., 2017).

Many epidemiological studies in the offspring exposed *in-utero* to the Dutch Famine (Lumey and Stein, 1997) or born with a low birth weight revealed anxiety disorders in the survivors as adults (Boyle et al., 2011). This led to creation of animal models with maternal calorie restriction, to determine the long-term impact on the adult brain. One such study performed by our group revealed that maternal and/or postnatal caloric restriction in rats caused hyperactivity in a subset of the adults (Tomi et al., 2013), However, in these studies we did not decipher any changes in adult brain SERT concentrations (Tomi et al., 2013). However, maternal calorie restriction demonstrated reduced serotonin and SERT concentrations in E19 mouse brains (Ye et al., 2021). In contrast, studies in mice reared on a high fat diet revealed depressive symptoms in the adult (Aslani et al., 2015), while *in-utero* exposure to a high fat diet led to a lower trend in E19 brain serotonin concentrations with no comparable change in SERT concentrations (Ye et al., 2021). Similar investigations are lacking during the postnatal phase of development when both serotonin and SERT in brain are known to reach peak concentrations.

Since a brain serotonin imbalance can alter emotions, it is known that more than 90% of serotonin in the body is synthesized in the gut by various serotonin-producing bacteria such as *Streptococcus* spp., *Enterococcus* spp., *Escherichia* spp., *Lactobacillus plantarum*, *Klebsiella pneumonia*, and *Morganella morganii* (Barandouzi et al., 2022). In addition, certain indigenous spore-forming microbes, specifically Clostridial species from the gut microbiota, produce metabolites that promote host serotonin biosynthesis in colonic enterochromaffin cells with changes in TPH1 and SERT expression, thereby impacting gastrointestinal motility and homeostasis (Yano et al., 2015). Further, Hsiao et al. demonstrated that the maternal microbiome also modulates fetal neurodevelopment (Vuong et al., 2020). In their studies, the maternal gut microbiome promoted fetal thalamocortical axonogenesis, likely by signaling of microbially modulated metabolites to neurons in the developing brain (Vuong et al., 2020). “Dysbiosis” of the maternal gut microbiome, in response to environmental changes such as infection, altered diet such as a high fat and high fructose diet during pregnancy, has been increasingly associated with abnormalities in the offspring’s brain function and behavior (Buffington et al., 2016). Thus altered gut microbiome can regulate the biosynthesis, release and reuptake of certain neurotransmitters including serotonin. This in turn can affect the host serotonin receptors and signaling pathways. Such disturbances in the gut microbiome related to early life exposures to certain diets may perturb the gut-brain axis toward affecting permanent axonal-circuitary. Such alterations can form the basis of perturbed neurobehaviors that span from heightened stress responses to full fledged presentations of neuropsychiatric disorders that become evident in the adult offspring.

Based on the collection of these prior observations, we hypothesized that both maternal calorie restriction and exposure to a high fat and high fructose diet during fetal and postnatal life would alter postnatal brain serotonin and/or SERT concentrations associated with a dysbiosis relevant to serotonin producing bacterial species. These changes would display a temporal pattern providing the gut-brain based mechanism for previously observed neuropsychiatric presentations in response to early dietary modifications.

## 2 Methods and materials

### 2.1 Animals

The study protocol was approved by the Animal Research Committee of the University of California Los Angeles (UCLA) in accordance with guidelines of the National Institutes of Health.

### 2.2 Animal models

Two-three month old timed pregnant Sprague–Dawley rats (Charles River Laboratories, Hollister, CA) were employed for creating the two models of (1) calorie restriction and (2) high fat/high fructose dietary exposures.

#### 2.2.1 Calorie restriction induced intra-uterine growth restriction with postnatal catch-up growth

##### 2.2.1.1 Prenatal and early postnatal studies

**Figure 1:**
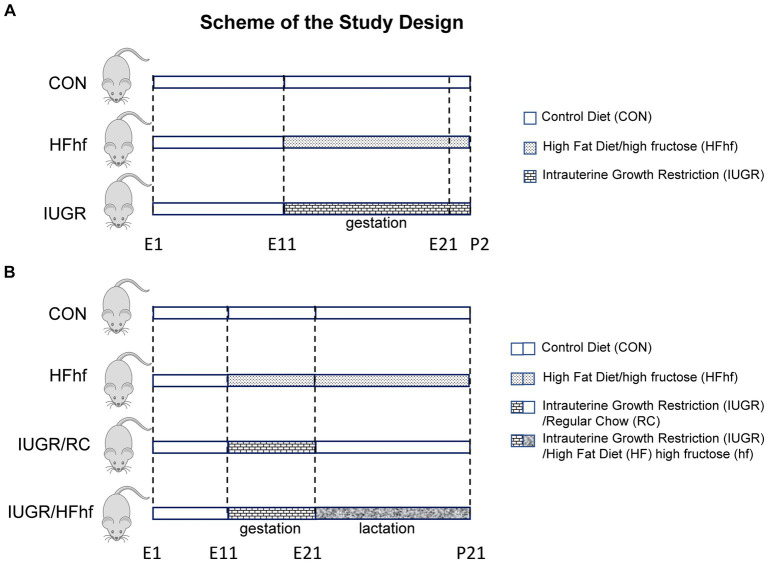
Scheme demonstrating the experimental design. **(A)** Early postnatal rats: Three groups were created. (1) Control (CON) mothers reared on regular chow diet, (2) High fat and high fructose (HFhf) mothers received HFhf diet with fructose in their drinking water from gestational and embryonic day 11 (E11) to P2, (3) The intrauterine growth restriction (IUGR) group mothers received reduced regular chow diet from gestational and embryonic day 11 to P2. **(B)** Late postnatal rats: Soon after birth, CON and HFhf groups were given the control diet or the HFhf diet, respectively, until postnatal day 21 (P21). On the other hand, the IUGR group was given either the regular control diet (RC) (IUGR/RC) or the HFhf diet (IUGR/HFhf) until postnatal day 21 (P21).

Pregnant rats that received 50% of their daily calorie intake (11 g/day) beginning from day 11 through day 21 of gestation (IUGR) versus *ad libitum* access to regular chow diet (22 g/day) (CON) were allowed to spontaneously deliver pups. High fat diet (HF; TD88137, Herlan Teklad Laboratories, Indianapolis, IN; composition 45%, fat 42% and protein 13%) with high fructose (hf) in the drinking water (25%; induces de-novo lipogenesis) (HFhf) was provided *ad libitum* from day 11 through day 21 of gestation, and this group of pregnant rats were also allowed to deliver pups spontaneously. These three groups had *ad libitum* access to drinking water (Figure 1A). At postnatal day 2 after the pups were delivered, they belonged to three experimental groups, namely CON, IUGR and HFhf. These P2 pups were deeply anesthetized and euthanized by inhalational 5% isoflurane, brains retrieved and snap frozen at −80°C until further analyses.

##### 2.2.1.2 Late postnatal studies

After delivery at P2, the lactating mothers in the *ad libitum* fed chow diet group were continued on regular chow diet and water with *ad libitum* access (CON). The group fed a high fat diet with high fructose in the drinking water prenatally were continued on the same diet *ad libitum* postnatally as well (HFhf) with *ad libitum* access to water. In contrast, the IUGR group was arbitrarily sub-divided into two postnatal groups, one that was continued on regular chow diet *ad libitum* (IUGR/RC) and the other exposed to a high fat+high fructose diet (IUGR/HFhf) *ad libitum* through the suckling phase, both IUGR sub-groups having *ad libitum* access to drinking water (Figure 1B). In these two IUGR subgroups, the prenatal to postnatal nutritional (calorie) mismatch was accomplished, respectively, by either providing *ad libitum* access to regular chow diet (IUGR/RC) or further maximized calories by introducing consumption of a high fat diet along with high fructose (IUGR/HFhf) postnatally. The lactating dams in the different groups were continued on their respective diets until weaning of the pups (Figure 1B). At postnatal day 21 (P21) four experimental groups (CON, IUGR/RC, IUGR/HFhf and HFhf) were created. The litter size was culled to eight at birth and maintained so throughout the suckling phase to ensure no postnatal inter-litter nutritional variability. P21 pups were deeply anesthetized and euthanized by inhalational 5% isoflurane, brains retrieved and snap frozen at −80°C until further analyses.

### 2.3 Antibodies

Rabbit anti-serotonin transporter (Millipore, Temecula, CA), mouse anti-vinculin and anti-β–actin (Sigma Chemical Co., St. Louis, MO.) antibodies were commercially obtained.

### 2.4 ELISA: brain serotonin

Brain serotonin concentrations were measured in duplicate within supernatants (50 μg protein measured by the Bio-Rad assay) of brain tissue homogenates by an ELISA kit (Serotonin High Sensitive ELISA, Eagle Biosciences, Nashua, NH) that has previously been validated (Yano et al., 2015). The measurements range from 0 to 100 pg./50 μg of brain homogenates. Sensitivity of the assay for serotonin is 0.39 pg./sample and the specificity is ~100%, with 0.22% for tryptamine, 0.025% for 5-methoxytryptamine, 0.0021% for 5-hydroxytryptophan, <0.001 for melatonin and 5-HIAA and < 0.0001 for L-tryptophan. The intra–assay coefficient of variation ranged from 6.6 to 8.7%.

### 2.5 Western blot analyses

Brain tissues were homogenized and sonicated in RIPA buffer (Thermo Scientific) or cell lysys buffer {(20 mM Tris–HCl (pH 7.5), 150 mM NaCl, 1 mM Na_2_EDTA, 1 mM EGTA, 1% NP-40, 1% sodium deoxycholate, 2.5 mM sodium pyrophosphate, 1 mM β-glycerophosphate, 1 mM Na_3_VO_4_, 1 μg/mL leupeptin (Pierce, Waltham, WA) with protease inhibitors (Thermo Scientific, Canoga Park, CA) including 2 mM PMSF}. The resulting homogenate was centrifuged at 10,000 g at a temperature of 4°C for 10 min, and the protein content in the supernatant was quantified by the Bio-Rad protein assay (Bio-Rad Laboratories, Irvine, CA) and subjected to Western blot analysis. Briefly, supernatants were solubilized in 4% SDS, separated by SDS-PAGE and transferred to nitrocellulose membranes. Membranes were washed with PBST X 3 for 5 min each and blocked in 3% bovine serum albumin for 1 h, followed by overnight incubation at 4°C with the specific primary antibody (anti-serotonin transporter antibody, 1:500 dilution [Millipore, Temecula, CA]). The membranes were stripped and incubated with vinculin (1: 5,000 dilution) as an internal loading control. Membranes were subsequently washed in PBST X 6 for 5 min each and incubated at room temperature over 45 min with the appropriate secondary horseradish peroxidase-conjugated antibody (Pierce, Waltham, WA). Prior to visualization of the protein bands using ECL plus kit (Pierce, Waltham, WA) under the Typhoon Scanner (GE Healthcare, Pasadena, CA) the membranes were washed again with PBST. The quantification of protein bands was performed by densitometry using Image Quant software (GE Healthcare, Pasadena, CA). The optical density was corrected for inter-lane loading variability using vinculin as an internal control, and expressed as a percent of respective CON values.

### 2.6 16S rDNA microbiota profiling

Microbiome profiling of gut bacteria was performed following the methods previously described by us (Maggiotto et al., 2023). Briefly, colonic fecal samples were collected from postnatal day 2 and day 21 old pups. DNA was extracted using Qiagen Powersoil kit. Libraries were generated according to methods adapted from Caporaso et al. (2011). The V4 region of the 16S rRNA gene was amplified with individually barcoded universal primers. PCR amplified products were purified and pooled in equimolar concentrations and sequenced using the Illumina MiSeq platform and 2 × 250 bp reagent kit for paired-end sequencing.

Sequencing analyses were performed as previously described (Maggiotto et al., 2023). Furthermore, multivariate associations between covariates (serotonin and SERT concentrations in brain) and microbiome features were performed using MaAsLin2 (an R package) (Mallick et al., 2021) which implements linear mixed-effects models.

### 2.7 Data analyses

Data are expressed as mean ± standard error of the mean. Sample size was predetermined by conducting a power analysis employing the Stat-mate software at a power of 80% and a *p* value of 0.05. Analysis of variance models were used to compare various treatment groups and differences between the two sexes. Inter-group and sex differences were determined *post-hoc* by the Tukey’s multiple comparison test. Statistical significance was established by Prism (7th edition) software and significance was assigned when the *p* value was <0.05.

## 3 Results

### 3.1 Early postnatal studies

The animal models created and sub-divided into four experimental groups are schematically depicted in Figures 1A, B.

### 3.2 Comparison of brain serotonin and SERT expression in response to prenatal and postnatal dietary changes

#### 3.2.1 P2 pups

**Figure 2:**
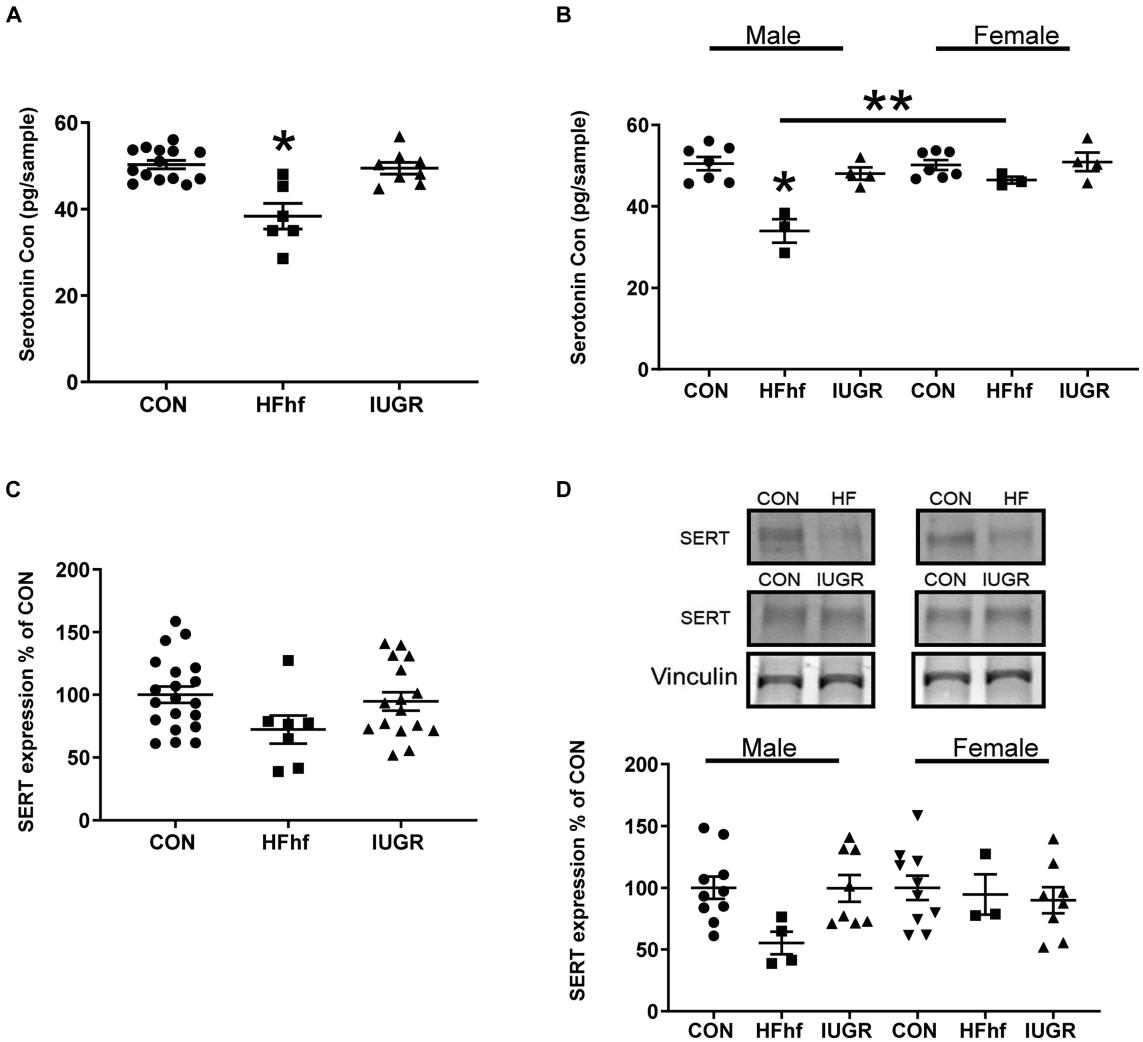
Brain serotonin and SERT protein concentrations in early postnatal day 2 (P2) rats. **(A)** Brain serotonin concentrations in combined males and females demonstrate a decrease by 24% in the high fat and high fructose (HFhf) diet group (*n* = 6) versus Control (*n* = 14), (One Way ANOVA F statistic, [df:2,22] = 15.84, *p* value = 0.00005, and Tukey’s *post-hoc* test *p = 0.00005). **(B)** Brain serotonin concentrations in separated males and females, demonstrate in male brain a decrease of 32% in HFhf (*n* = 3) vs. CON (*n* = 6), (Two Way ANOVA F statistic [df:2,22] = 15.84, *p* value = 0.0001, and Tukey’s *post-hoc* test *p = 0.000007), with no difference in females. There is no difference in females between HFhf and CON (Tukey’s *post-hoc* test *p* = 0.718). A significant difference was observed in serotonin concentrations between male and female HFhf groups, with females showing ~24% higher concentrations than males (Tukey’s *post-hoc* test **p = 0.006). **(C)** SERT expression when males and females were combined trended to a decrease by 28% in HFhf (*n* = 7) vs. CON (*n* = 20), (One Way ANOVA F statistic [df:2,40] = 2.351, *p* = 0.108). **(D)** When the sexes were separated, while again only a trend toward a decline of ~45% in SERT concentrations occurred in the male HFhf (*n* = 4) vs. the male CON (*n* = 10), no significant difference was found (Two Way ANOVA F statistic [df:2,37] = 2.38, *p* value = 0.106). No inter-group differences were seen in the females, and no male versus female differences were noted either (Two way ANOVA F statistic [df:1,37] = 0.08, *p* value = 0.771), nor group X sex interaction differences (Two way ANOVA F statistic [df:2,37] = 1.71, *p* value = 0.193). Further, there is no difference in brain serotonin and SERT concentrations in the IUGR group versus control whether the sexes were combined or separated. Data are shown as mean ± SEM.

In the P2 rat pup when males and females were combined, brain serotonin concentrations decreased by 24% (One Way ANOVA F statistic, [df:2,22] = 15.84, *p* value = 0.00005, and Tukey’s *post-hoc* test *p* = 0.00005) in the high fat diet with high fructose (HFhf) exposed group (*n* = 6) when compared to Control (CON; *n* = 14) (Figure 2A). When the sexes were separated, specifically, in male brains (Figure 2B) serotonin decreased by 32% (Two Way ANOVA F statistic [df:2,22] = 15.84, *p* value = 0.0001, and Tukey’s *post-hoc* test *p* = 0.000007) in HFhf (*n* = 3) versus CON (*n* = 6). There was no significant difference observed in females between HFhf and CON groups (Tukey’s *post-hoc* test *p* = 0.718). However, a significant difference in brain serotonin concentrations between males and females within the HFhf group emerged (Tukey’s *post-hoc* test *p* = 0.006), with females showing ~24% higher concentrations than males. Again when the sexes were combined, brain SERT protein concentrations trended to a decrease by 28% (One Way ANOVA F statistic [df:2,40] = 2.351 *p* = 0.108) in HFhf (*n* = 7) versus CON (*n* = 20) (Figure 2C). However, no significant inter-group differences were seen (Two Way ANOVA F statistic [df:2,37] = 2.38, *p* value = 0.106), nor male versus female sex differences observed (Two way ANOVA F statistic [df:1,37] = 0.08, *p* value = 0.771) with a lack of group X sex interaction difference (Two way ANOVA F statistic [df:2,37] = 1.71, *p* value = 0.193). The IUGR P2 males and females, both demonstrated no changes in either brain serotonin or SERT concentrations when compared to age- and sex-matched controls (Figures 2B,D).

#### 3.2.2 P21 pups

**Figure 3:**
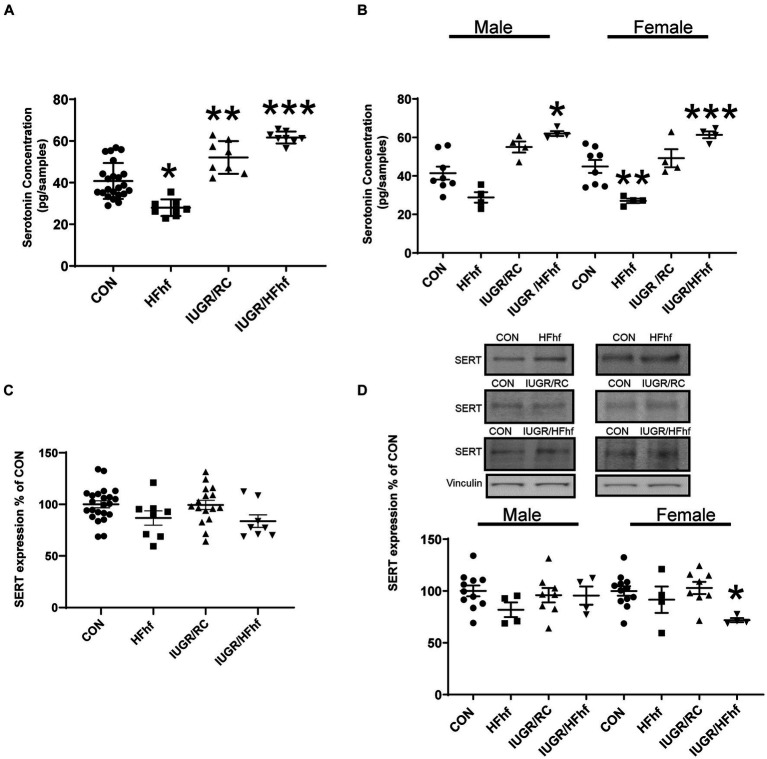
Brain serotonin and SERT protein concentrations in late postnatal day 21 (P21) rats. **(A)** Brain serotonin concentration in combined males and females decreased by 30% in HFhf (*n* = 8), (One Way ANOVA F statistic [df:3,43] = 34.44, *p* value <0.001, and Tukey’s *post-hoc* test *p = 0.0005) but increased by 30% in IUGR/RC (regular chow diet) (*n* = 8). (Tukey’s *post-hoc* test **p = 0.002) and by 53% in IUGR/HFhf groups (*n* = 8) vs. CON (*n* = 23), (Tukey’s *post-hoc* test ***p < 0.0001). **(B)** When the sexes were separated, serotonin trended to decrease by 32% in male HFhf (*n* = 4), (Two Way ANOVA F statistics [df:3,32] = 29.48, *p* value <0.00001, and Tukey’s *post-hoc* test *p* = 0.146) while trending to increase by 34% in male IUGR/RC (*n* = 4; Tukey’s *post-hoc* test *p* = 0.095) and increasing by 51% in male IUGR/HFhf (*n* = 4) vs. CON (*n* = 8; Tukey’s *post-hoc* test *p = 0.0005). In the female, brain serotonin decreased by 40% in HFhf (*n* = 4; Tukey’s *post-hoc* test **p = 0.010), while increasing by 36% in IUGR/HFhf (*n* = 4; Tukey’s *post-hoc* test ***p = 0.02259) vs. the sex-matched CON (*n* = 8). **(C)** When the sexes are combined, no difference in SERT expression was seen in all groups. **(D)** Upon separation of males from females, while no differences were seen in the male groups, SERT concentration only decreased by 28% in the female **(D)** IUGR/HFhf (*n* = 4) vs. CON (*n* = 8; Two Way ANOVA F statistic [df:3,24] = 3.54, *p* value = 0.03, and Tukey’s *post-hoc* test *p = 0.036) in IUGR. No male versus female differences were seen even within the IUGR/HFhf group (Tukey’s *post-hoc* test *p* = 0.11) nor any differences in the group X sex interaction. Data are shown as mean ± SEM.

At P21, male and female combined rat brain serotonin concentrations remained decreased by 30% (One Way ANOVA F statistic [df:3,43] = 34.44, *p* value <0.001, and Tukey’s *post-hoc* test *p* = 0.0005) in HFhf (*n* = 8), while brain serotonin concentrations increased by 30% (Tukey’s *post-hoc* test *p* = 0.002) in IUGR/RC (regular chow diet) (*n* = 8) and 53% in (Tukey’s *post-hoc* test *p* < 0.0001) IUGR/HFhf diet (*n* = 8) versus CON (*n* = 23) (Figure 3A). Upon separation of sexes, particularly in the male, brain serotonin trended to a decrease by 32% (Two Way ANOVA F statistics [df:3,32] = 29.48, *p* value <0.00001, and Tukey’s *post-hoc* test *p* = 0.146) in HFhf (*n* = 4) and trended to increase by 34% (Tukey’s *post-hoc* test *p* = 0.095) in IUGR/RC (*n* = 4), being higher by 51% (Tukey’s *post-hoc* test *p* = 0.0005) in IUGR/HFhf (*n* = 4) versus CON (*n* = 8). Therefore, statistically significant difference was only observed between male CON and IUGR/HFhf groups. In the female counterpart, brain serotonin decreased by 40% (Tukey’s *post-hoc* test *p* = 0.010) in HFhf (*n* = 4) but increased by 36% (Tukey’s *post-hoc* test *p* = 0.02259) in IUGR/HFhf (*n* = 4), with no change in IUGR/RC when compared to CON (*n* = 8) (Figure 3B). In contrast, no differences were seen in SERT protein concentrations in all P21 brains when combined (Figure 3C). Upon separation of the sexes, while no changes in male brains were observed, in the female brain SERT expression decreased by 28% (Two Way ANOVA F statistic [df:3,24] = 3.54, *p* value = 0.03, and Tukey’s *post-hoc* test *p* = 0.036) in IUGR/HFhf (*n* = 4) versus CON (*n* = 8) (Figure 3D). No male versus female serotonin or SERT differences emerged, nor any group X sex interaction differences were noted.

#### 3.2.3 Association between brain serotonin/SERT and gut dysbiosis

**Figure 4:**
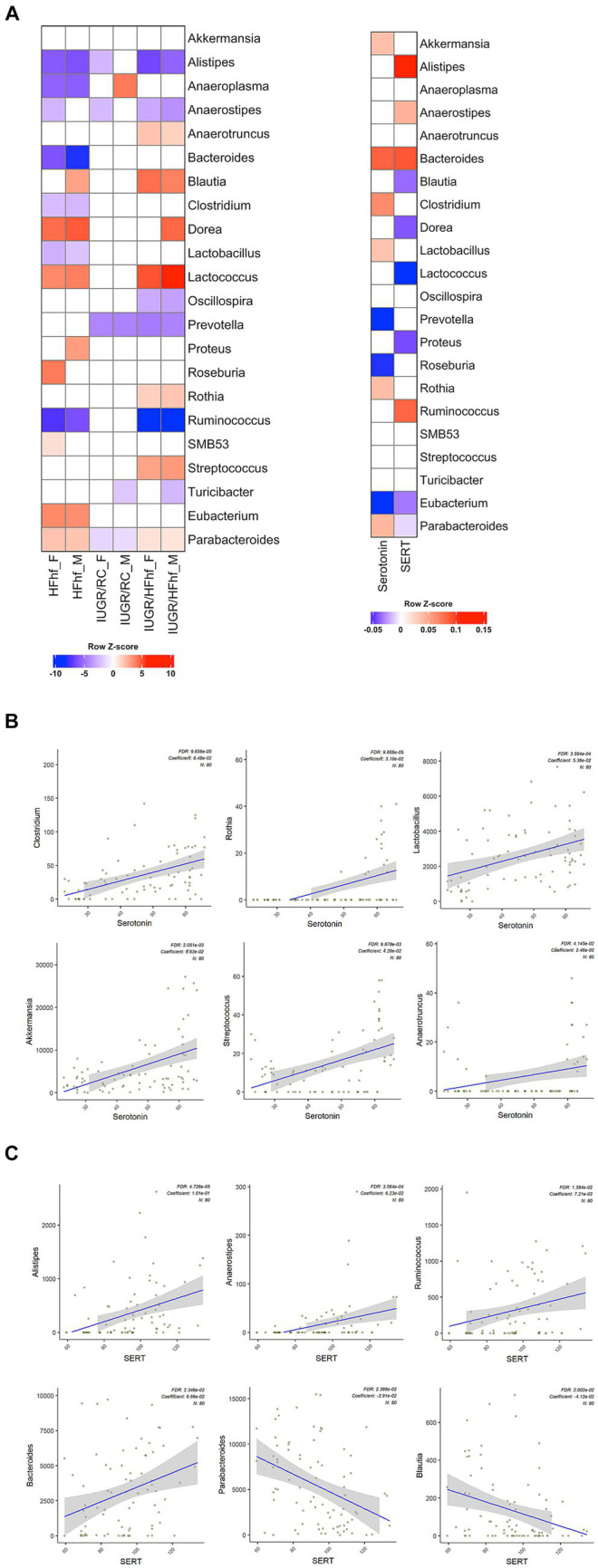
Association between gut bacterial genera and brain serotonin or SERT in late postnatal day 21 rats. **(A)** Heatmaps showing differential abundance of gut bacterial genera among the various dietary experimental groups (HFhf, IUGR/HFhf, and IUGR/RC versus CON) stratified by sex (male = M, female = F) (left panel) and their relation to brain serotonin or SERT (right panel). A color gradient demonstrates the significant shifts in the abundance of gut microbial genera, with red indicating enrichment and blue indicating depletion. MaAsLin2 was employed adjusting for the effects of diet and sex with default parameters and the CON dietary group served as a reference for associations with the other three different dietary intervention groups. **(B, C)** Scatter plots depicting associations between individual gut bacterial genera and brain Serotonin **(B)** and SERT **(C)** concentrations. This association was calculated using a MaAsLin2 model without adjusting the effects of diet and sex. Coefficients depict the strength and direction of the observed associations. FDR represents the corrected significance and “N” indicates the total sample size employed in the analyses.

Gut bacteria can influence brain physiology by generating various neurochemicals used by brain and specific species of gut bacteria can produce small molecule neurotransmitters, i.e., serotonin, dopamine, norepinephrine, epinephrine, GABA and acetylcholine (Strandwitz, 2018; Barandouzi et al., 2022). To determine the relationship between gut microbiome and brain serotonin or serotonin transporter (SERT) we performed linear regression analysis using MaAslin2 and our data demonstrated significant correlation between serotonin or SERT and fecal/gut microbiome. We did not observe significant associations at P2 (Supplementary Figure 1). However, at P21 associations between different gut bacterial genera and brain serotonin concentrations emerged. Furthermore, associations between SERT and certain gut microbiome related bacterial genera were evident. The results of these analyses have been summarized in Table 1 and Figure 4A.

**Table 1:** Associations between brain serotonin/SERT and certain gut bacterial genera (microbiome) isolated from the three different dietary experimental groups (HFhf, IUGR/HFhf, IUGR/RC versus CON).

Gut bacterial genera	Brain serotonin/SERT	Fold change	*p* value	Q value	Groups with changes and directionality
*Alistipes*	SERT	1.105979	6.39E-07	4.73E-05	HFhf, IUGR/HFhf↓
*Anaerostipes*	SERT	1.064273	2.43E-05	0.000359	HFhf, UGR/HFhf↓
*Lactococcus*	SERT	0.94102	0.00013	0.001602	HFhf, IUGR/HFhf↑
*Eubacterium*	SERT	0.968396	0.001448	0.009878	HFhf, IUGR/HFhf↑
*Proteus*	SERT	0.961326	0.002296	0.013838	HFhf, IUGR/HFhf↑
*Ruminococcus*	SERT	1.05181	0.004056	0.020008	HFhf, IUGR/HFhf↓
*Blautia*	SERT	−0.959605	0.004329	0.020023	HFhf, IUGR/HFhf↑
*Bacteroides*	SERT	1.068868	0.005389	0.023459	HFhf↓
*Parabacteroides*	SERT	−0.961667	0.005803	0.023858	HFhf, IUGR/HFhf↑
*Dorea*	SERT	−0.953993	0.008156	0.031765	HFhf, IUGR/RC, IUGR/HFhf↑
*Anaerotruncus*	SERT	−0.980914	0.010549	0.037173	IUGR/HFhf↑
*Clostridium*	Serotonin	1.066998	3.15E-06	9.66E-05	IUGR/HFhf↑
*Rothia*	Serotonin	1.031466	3.91E-06	9.66E-05	IUGR/HFhf↑
*Lactobacillus*	Serotonin	1.055032	1.96E-05	0.000359	HFhf↓
*Akkermansia*	Serotonin	1.068552	0.000194	0.002051	IUGR/HFhf↑
*X.Eubacterium.*	Serotonin	−0.952101	0.000228	0.002108	IUGR/HFhf↑
*Prevotella*	Serotonin	−0.939622	0.000945	0.007771	IUGR/RC↓
*Streptococcus*	Serotonin	1.043542	0.001468	0.009878	IUGR/HFhf↑
*Bacteroides*	Serotonin	1.097267	0.00307	0.016225	HFhf↓
*Sutterella*	Serotonin	1.040513	0.008622	0.031903	NA
*Anaerotruncus*	Serotonin	1.024905	0.012323	0.041451	IUGR/HFhf↑
*Roseburia*	Serotonin	−0.954285	0.013764	0.044285	HFhf↓

Arrows demonstrate the change in direction (increase ↑, decrease ↓).

Briefly, positive associations between brain serotonin and *Clostridium, Rothia, Lactobacillus, Akkermansia, Streptococcus* and *Anaerotruncus* in the gut was detected (Figure 4B). *Lactobacillus* and *Streptococcus* (Yaghoubfar et al., 2020) species have previously been reported to produce serotonin in the gut. In an *in-vitro* study *Akkermansia* species were also reported to promote secretion of serotonin in the gut (Yaghoubfar et al., 2020). Simultaneously, we observed significant positive correlation between brain SERT concentrations and fecal abundance of *Alistipes, Anaerostipes, Ruminococcus* and *Bacteroides* in P21 groups (Figure 4C). Brain SERT concentrations were negatively correlated with fecal *Parabacteroides* and *Blautia*. We also identified sex specific changes in genera that were associated with serotonin or SERT (Figure 4A). Notably, *Dorea* was increased in male not female IUGR/HFhf group but *Ruminococcus* was reduced in males and females in the IUGR/HFhf group. These genera were negatively and positively associated with brain SERT, respectively, which is consistent with the lack of decreased SERT in male IUGR/HFhf while females showed significantly reduced SERT. *Alistipes* and *Anaerostipes* had distinct differential abundance patterns between male and female pups in the IUGR/RC group and *Anaerostipes*, *Blautia*, *Proteus*, and *Roseburia* differed by sex in the HFhf group.

## 4 Discussion

In our present study, we have demonstrated an imbalance between brain serotonin and SERT concentrations early in life in response to two different maternal dietary modifications. Serotonin being necessary for normal brain development, these observed changes have the ability to affect brain plasticity and adaptation of the serotonergic neural networks during their formative stages of embryonic and postnatal development, with a lasting impact on adult neurobehavioral expression. In addition, to these early life changes, we have observed sex-specific changes as early as during the suckling phase of postnatal development. We and others have conducted many postnatal studies with an impact on the adult phenotype in the rat (Cha et al., 1987; Garg et al., 2010, 2012, 2013; Dai et al., 2012; Shin et al., 2012), a species that displays exemplary maternal instincts and nurturing capabilities.

To ensure nutritional consistency, it is imperative to maintain the same litter size throughout the suckling phase. The previously characterized rat model of maternal calorie restriction producing intra-uterine growth restriction (Dai et al., 2012; Garg et al., 2012; Shin et al., 2012) was instituted prenatally at a time in gestation when denovo synthesis of serotonin occurs in the brain (Gaspar et al., 2003). In the hyper-caloric group the rats received high fat + high fructose in their diet to reflect the consumption of high fat and high carbohydrates in the Western population. Soon after birth (P1-P2), the mothers in both the IUGR and the HFhf groups were placed on HFhf diet and compared to controls reared on a regular chow (RC) diet. These mothers’ transmitted their metabolic changes in response to dietary modifications via their milk to their suckling offspring (Nicholas and Hartmann, 1991). In response to intra-uterine exposure to a maternal caloric restricted environment, the IUGR offspring revealed no changes in the immediate postnatal brain serotonin and SERT concentrations, while the male HFhf offspring displayed a diminution in both molecules. These observations demonstrate differences in the maternal dietary impact upon embryonic (via the placenta) and postnatal (via mother’s milk) brain 5-HT-SERT axis.

Further examination at P21 after having been exposed to the same postnatal dietary changes for a longer period of time, led to a persistent diminution of brain serotonin concentrations in both the HFhf male and female offspring, while the SERT concentrations were the same as control concentrations. This misalignment between serotonin-SERT may reflect lower synaptic availability of serotonin. In contrast, the IUGR offspring that was reared by a mother switched to a regular chow diet, demonstrated higher than normal in male and no change in female brain serotonin concentrations. While no differences were observed with SERT in the males, a reduction in SERT was evident in the female IUGR/HFhf group versus the other three groups. Thus, at this postnatal age, these changes set the stage for availability of higher serotonin concentrations in the synaptic cleft in both sexes via differing mechanisms.

In addition to these postnatal brain serotonin-SERT changes, significant alterations in the gut microbial constituents were seen at this developmental stage. These changes consist of a significant decrease in *Bacteroides* species within both the male and female sexes in certain groups exposed to maternal dietary modifications. *Clostridium* species were observed to increase in the three male groups while *Streptococcus* species increased in the IUGR/HFhf group alone versus the controls. Clostridium species is involved in serotonin production (Kuley et al., 2011) as well. Brain SERT concentrations which affect the availability of serotonin were also associated with changes in specific gut microbial genera in a sex-specific manner. Such changes in serotonin or other metabolite producing or regulating microbes can lead to a disequilibrium of the ecosystem of the host gut. This in turn may contribute toward dysregulation locally in serotonin or other metabolite production. Such perturbations have the propensity of altering host processes that impact circulatory concentrations and in some cases the neural networks including that of serotonin. These findings within the postnatal gut microbiome secondary to maternal dietary changes, along with the dysregulation of brain serotonin-SERT system, gives credence to the concept of the gut microbiome-brain axis. In fact changes in the maternal gut microbiome in mice have been demonstrated to influence prenatal neurodevelopment (Vuong et al., 2020, 2021). Our correlative observations between specific gut microbiota and brain serotonin/SERT concentrations, albeit descriptive, may support the influence of the gut microbiome during the critical window of prenatal and postnatal serotonergic neurodevelopment.

While we did not assess all the dietary groups, limited follow-up long term in the 180d adult male and female offspring temporally demonstrated a lack of persistence of these early life changes in brain serotonin in the HFhf and SERT concentrations in the IUGR-HFhf and HFhf fed groups when compared to respective controls (unpublished). A similar finding was also seen in the IUGR/RC male and female offspring in our previous study (Tomi et al., 2013). Thus it appears that even if the adult brain concentrations of serotonin and SERT may normalize despite ongoing exposure to the respective dietary modifications, the perturbations induced during the critical embryonic/postnatal window of plasticity in development may alter multiple aspects of neural wiring that may have long lasting functional effects upon the brain (Daws and Gould, 2011).

While in this study we did not undertake neurobehavioral studies, many other investigators have studied the neurobehavior of the adult IUGR offspring or high fat exposed offspring, and observed anxiety and hyperactivity in the former (Lahti et al., 2010; Mikaelsson et al., 2013; Tomi et al., 2013) and depressive behaviors in the latter (Contu and Hawkes, 2017). While there may be multiple factors contributing toward this ultimate neurobehavioral phenotype, our studies demonstrate a major role of maternal diet upon the chiseling of this ultimate phenotype. In addition, despite the normalization of either serotonin or SERT in the adult brain, various other perturbations related to activation of enhancers/modifiers, suppression of endogenous inhibitors, or other epigenetic factors can alter the serotonin-SERT pathway resulting in altered availability of serotonin for neurotransmission (Daws and Gould, 2011; Brummelte et al., 2017). Our observations where maternal dietary modifications perturb the 5-HT-SERT balance is reminiscent of the impact of drugs such as selective serotonin reuptake inhibitors (SSRI) that increase the availability of synaptic serotonin for neurotransmission. Maternal SSRIs can easily cross the placenta and alter fetal brain serotonin availability (Olivier et al., 2013), akin to what we observed in the postnatal IUGR offspring.

Genetic mutations of the serotonin receptor isoforms (5-HT1B and 5-HT1D) have led to changes in fetal neural circuitry causing aberrant neuro-behavior (Bonnin et al., 2007; Bonnin and Levitt, 2011). Non-genetic environmental alterations in either serotonin or SERT or both impact the synaptic serotonin availability and function. Particularly during embryonic development, serotonin availability is necessary for neurogenesis, migration and axonal development (Gaspar et al., 2003). During the postnatal stage, when brain serotonin and SERT surge, increasing dendritic-axonal networks and thereby neural connections and circuitry are established along with ongoing pruning (Daubert and Condron, 2010; Lesch and Waider, 2012). We have shown that dietary environments can cause an early 5-HT-SERT imbalance with the possibility of deranging neural/axonal development and circuitry. These derangements come with the propensity of long-term implications that adversely affect plasticity and adult neuro-behavior (Shah et al., 2018). More recently, mutations of SERT have been implicated in autism spectrum disorders (Muller et al., 2016; Siemann et al., 2017), and circulating maternal serotonin concentrations have been associated with ASD in their offspring (Montgomery et al., 2018). However, serotonin fails to cross the placenta (Bonnin et al., 2011), suggesting other indirect effects of these changes having implications for the developing embryo. It is known that males predominate in developing ASD (Loomes et al., 2017), while females have a higher incidence of anxiety (McLean et al., 2011). We have observed a sex-specific effect of changes in serotonin, with the male predominating in particular. However, longer duration of exposure to dietary modifications end up affecting both the males and females as observed at P21. It is important to decipher the mechanism of action between diet and 5-HT-SERT pathway. While one of the mechanisms tying the dietary modifications to the neural serotonin-SERT connectivity may involve the gut microbiome as the intermediary, other dietary modified enhancers/inhibitors or epigenetic/nutrigenomics may also contribute requiring future mechanistic investigations (Daws and Gould, 2011; Brummelte et al., 2017). Further the impact of the X versus the Y chromosome upon these mediating molecular mechanisms needs unraveling.

In conclusion, we have demonstrated that prenatal maternal dietary modifications (either low or high calories) alter postnatal brain serotonin and/or SERT concentrations in a sex-specific manner, with the male offspring being primarily affected during the immediate early postnatal stage. With ongoing exposure to the altered dietary environment, both males and females are ultimately affected. The IUGR offspring created in response to maternal caloric restriction demonstrates a reduction in serotonin with concomitant diminution of SERT which can be restituted with a normal postnatal diet, only to overshoot the normal with a prenatal to postnatal nutritional mismatch, namely postnatal exposure to a high fat and high fructose diet. In contrast, a high fat and high fructose diet prenatally and postnatally creating a nutritional match led to a reduction in serotonin with no change in SERT, particularly in the male offspring. Thus reduced calorie intake by mothers can increase serotonin availability, while increased calorie intake decrease serotonin availability during this critical window of development. These changes may underlie the development of anxiety and hyperactivity in the adult IUGR offspring (Lahti et al., 2010; Mikaelsson et al., 2013) and depression in the offspring exposed to early high fat and high fructose nutritional environment (Contu and Hawkes, 2017). In all cases, the molecular mechanisms tying early introduction of dietary modifications with the developing offspring’s brain neurotransmitters is of significant importance necessary for unraveling the pathogenesis of neurodevelopmental and mental health disorders. The resulting gut dysbiosis induced by the maternal dietary modifications likely underlies the brain-gut axis reliant changes seen in the developing postnatal brain. Future mechanistic studies are warranted toward unraveling mechanisms required for developing targeted therapeutic measures.

## Data availability statement

The datasets presented in this study can be found in online repositories. This data can be found in the Gene Expression Omnibus accessible through GEO Series accession number GSE220146.

## Ethics statement

The animal study was approved by UCLA Animal Research Committee. The study was conducted in accordance with the local legislation and institutional requirements.

## Author contributions

XY: Formal analysis, Investigation, Methodology, Validation, Writing – original draft, Writing – review & editing. SG: Data curation, Formal analysis, Investigation, Methodology, Software, Validation, Writing – review & editing. BS: Investigation, Methodology, Supervision, Validation, Writing – review & editing. AG: Methodology, Writing – review & editing. LM: Methodology, Writing – review & editing. JJ: Data curation, Software, Validation, Writing – review & editing. SD: Conceptualization, Data curation, Formal analysis, Funding acquisition, Investigation, Methodology, Project administration, Resources, Software, Supervision, Validation, Visualization, Writing – original draft, Writing – review & editing.
