# Supplementary figures and images for: Brain serotonin and serotonin transporter expression in male and female postnatal rat offspring in response to perturbed early life dietary exposures
Source: Front Neurosci. 2024 Mar 21;18:1363094. doi: 10.3389/fnins.2024.1363094 (PMC10991790; doi:10.3389/fnins.2024.1363094)

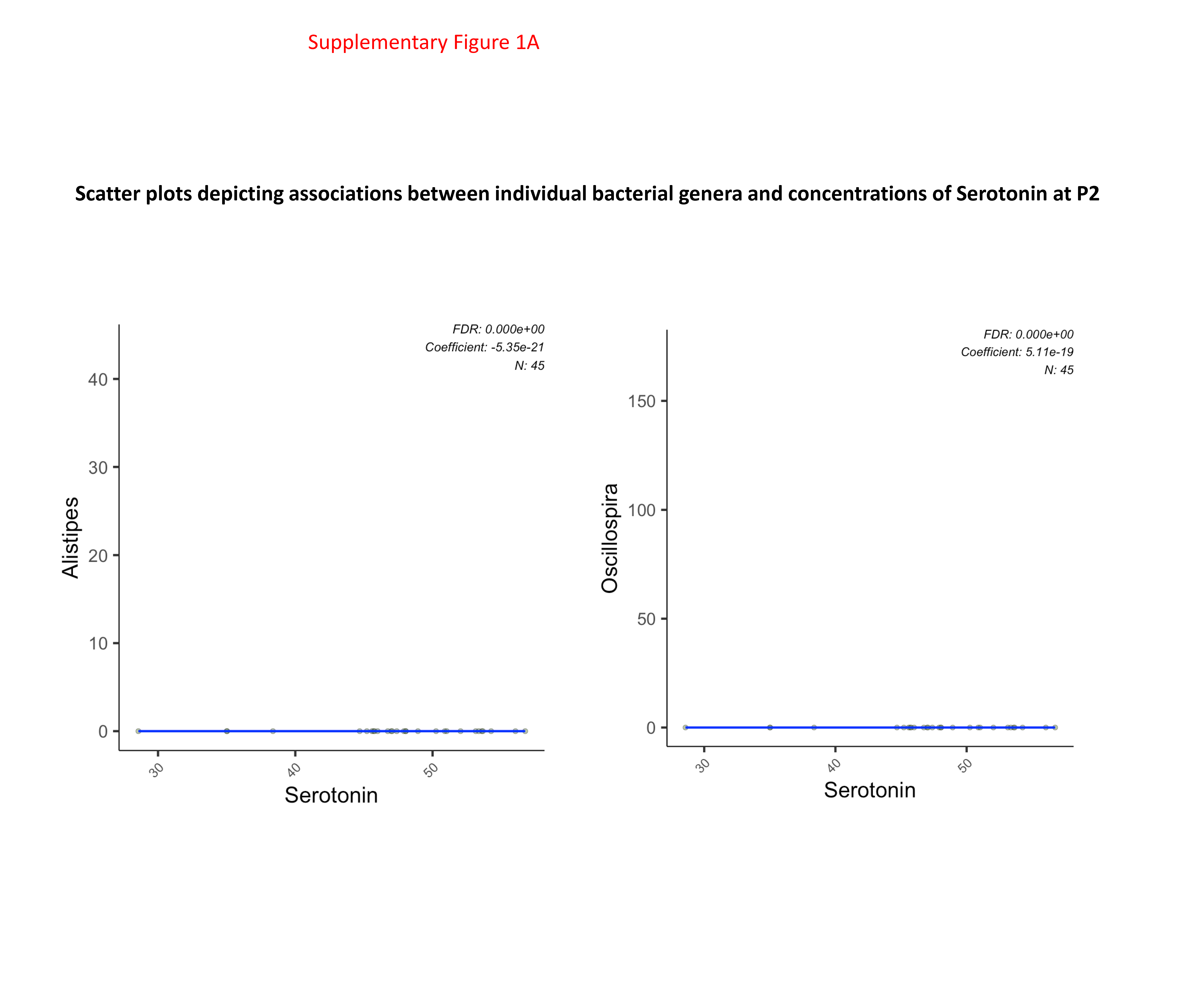

Supplement: Supplementary file 1 [file Image_1.TIFF]

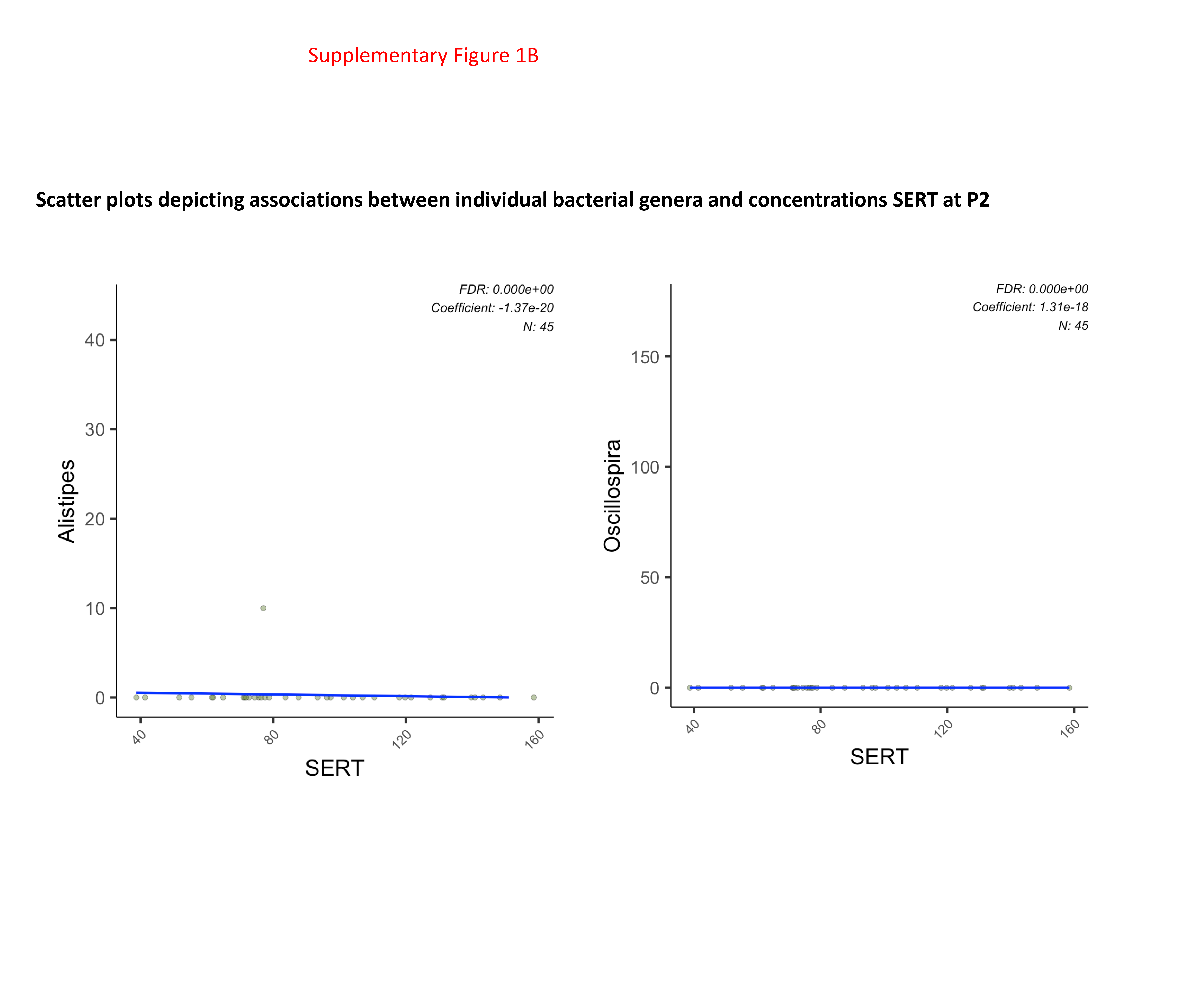

Supplement: Supplementary file 2 [file Image_2.TIFF]
